# Correction: Bakuchiol – a natural meroterpenoid: structure, isolation, synthesis and functionalization approaches

**DOI:** 10.1039/d2ra90033e

**Published:** 2022-04-07

**Authors:** T. P. Adarsh Krishna, Baldev Edachery, Sunil Athalathil

**Affiliations:** R & D Division, Sreedhareeyam Farmherbs India Pvt. Ltd Ernakulam (Dist.) Kerala India-686 662 adarshkrishnatp@gmail.com

## Abstract

Correction for ‘Bakuchiol – a natural meroterpenoid: structure, isolation, synthesis and functionalization approaches’ by T. P. Adarsh Krishna *et al.*, *RSC Adv.*, 2022, **12**, 8815–8832, DOI: 10.1039/D1RA08771A.

The authors regret that the first sentence in Section 2 (Occurrence and isolation) contained errors. The correct sentence is the following:

“Bakuchiol (Syn. Chiba) was first extracted from the seeds of *Psoralea corylifolia* L. Medik (Bakuchi, now *Cullen corylifolium* L.) by a team of scientists at the National Chemical Laboratory (NCL), Pune, India.”

The Royal Society of Chemistry apologises for these errors and any consequent inconvenience to authors and readers.

## Supplementary Material

